# Clinical characteristics of primary pars plana vitrectomy combined with air filling for rhegmatogenous retinal detachment

**DOI:** 10.1038/s41598-022-12154-z

**Published:** 2022-05-12

**Authors:** Changzhong Xu, Jianhua Wu, Yanzi Li, Rui Zhang, Chao Feng

**Affiliations:** 1 Department of Ophthalmology, The Second Xiangya Hospital, Central South University, Changsha, China; 2 Aier Eye Hospital of Wuhan University, No.481 Zhongshan Road, Wuchang District, Wuhan, China

**Keywords:** Diseases, Medical research, Risk factors

## Abstract

To detect the prognostic factors associated with initial reattachment after primary pars plana vitrectomy (PPV) with air tamponade for rhegmatogenous retinal detachment (RRD). We retrospectively reviewed 92 eyes of 92 patients with RRD. All eyes underwent PPV with air tamponade and a follow-up of at least 6 months. Initial anatomical success was defined as reattachment of the retina by a single operation. We performed univariate analysis to detect the presence of any difference between eyes with a successful initial reattachment and those that failed. We also performed multivariate logistic regression analysis to assess the influence of each preoperative factor on initial success. The rate of initial reattachment success was 93.5%. The percentage of retinal detachment involving the inferior quadrants in the initial success group was less than that in the initial failure group, and the difference was statistically significant (P = 0.043). There were no significant differences noted for other factors, such as symptom duration (P = 0.078) or location of retinal breaks (P = 0.065). Multiple logistic regression analysis using preoperative factors indicated that older age (odds ratio, 0.90; 95% confidence interval, 0.82–0.97; P = 0.010) and non-involvement of inferior quadrants (odds ratio, 9.90; 95% confidence interval, 1.36–71.92; P = 0.023) were significantly associated with initial success. PPV combined with air may be an effective treatment for some simple RRDs (proliferative vitreoretinopathy [PVR] grade ≤ C1). Non-involvement of the inferior quadrants and older age at presentation are associated with a greater likelihood of anatomic success. The volume of air in the eye after surgery is also very important, which may also affect the reduction of retinal detachment.

## Introduction

Rhegmatogenous retinal detachment (RRD) is one of the most serious ocular diseases threatening vision. The treatment of RRD mainly includes pneumatic retinopexy, scleral buckling and pars plana vitrectomy (PPV)^1–4^. Due to the ease of application and the satisfactory visualization of the peripheral retina with scleral indentation and wide-angle viewing systems, PPV is becoming increasingly popular for the treatment of RRD^5^. For noncomplex retinal detachment, the most widely selected intraocular filler materials are long-acting gases (including 18%-20% SF6, 12%-14% C3F8, etc.)^6,7^. However, long-term gas absorption is slow, and long-term gas filling makes the surgical eye unable to regain vision in a short time, especially for patients with poor vision in nonsurgical eyes, which affects their postoperative daily life. Second, the half-life of expansive gas is longer, and the compliance requirements for patients in a long-term prone position after an operation are also higher. Moreover, in recent years, the expansion of gases has been limited in China. Therefore, silicone oil and filtered air have become two options for such patients. However, choosing silicone oil as a filler agent may cause complications such as secondary glaucoma, cataract formation and corneal complications, and silicone oil requires a second operation for removal after oil tamponade, which increases the economic burden on patients.

Previous studies have shown that adhesion of the retina and choroid occurs within 24 h after retinal laser photocoagulation, and then the liquid in the vitreous cavity no longer enters the retina through the hole. Although the half-life of air is short, the vitreous cavity is almost filled with air in a short time after vitrectomy, resulting in a large contact arc at the retinal interface. In the first week after vitrectomy, the air contact area at the retinal interface is large enough to enhance the absorption of subretinal fluid and the adhesion of retinal pigment epithelium caused by intraoperative laser retinal fixation^3,8–10^. Several studies have reported satisfactory results with air tamponade^3,9,11–13^. However, few relevant studies have evaluated the preoperative factors affecting the success rate of vitrectomy combined with air filling to treat uncomplicated RRD.

This study retrospectively analysed the effect of PPV combined with air filling in the treatment of uncomplicated RRD. At the same time, a multivariate logistic regression model was conducted on the preoperative factors affecting retinal reduction after the initial operation of PPV to evaluate its impact on the initial reattachment rate.

## Materials and methods

### Patients

We retrospectively reviewed the medical records of patients with primary RRD who underwent 23G or 25G PPV and air tamponade at Aier Eye Hospital of Wuhan University between August 2015 and September 2019. We excluded cases treated by scleral buckling with or without PPV or choosing silicone oil or expansion gas as a tamponade agent. Patients who had a less than six months follow-up time after the primary operation and had a history of intraocular surgery were excluded, except for cataracts. The institutional review board of Aier Eye Hospital of Wuhan University approved the study protocol (2021IRBLW03), and the protocol complied with the tenets of the Declaration of Helsinki. All patients signed informed consent forms before surgery.

### Data collection

All patients underwent comprehensive ophthalmic examinations during the follow-up period, including (1) best-corrected visual acuity (BCVA), using a Snellen visual acuity chart. (2) The intraocular pressure (IOP) was measured by a noncontact tonometer. (3) The lens status, anterior segment, and fundus conditions were evaluated by a slit-lamp biomicroscope and an indirect ophthalmoscope. (4) The extent of retinal detachment, whether it was combined with choroidal detachment, the essential characteristics of retinal breaks (such as location, type, size, and the number of holes), the presence of degenerative retinal areas and the classification of postoperative proliferative vitreoretinopathy (PVR) were recorded from preoperative A- and B-ultrasonography, ultrasound biomicroscopy (UBM), fundus colour photography or scanning laser ophthalmoscopy (SLO) (Optos 200Tx; Optos PLC, Dunfermline, Scotland). (5) According to the surgical records, we reconfirmed the details, including retinal breaks, range of retinal degeneration, quadrants involved in retinal detachment, and macular status. Furthermore, additional data were also collected from the records, e.g., endolaser coagulation, scleral cryotherapy, iatrogenic holes, incision sutures, whether heavy water (perfluoro-n-octane, PFO) was used, whether cataract extraction was combined, and intraoperative complications. The gas volume, BCVA, IOP, slit-lamp examination, and fundus examination on the first day, one week, one month, three months, six months and the last follow-up after the operation were obtained from the patients’ medical records. The primary outcome measures were the single operation retinal reattachment rate, final retinal reattachment rate, BCVA, and surgical complications. We defined the success of the initial surgery as retinal reattachment with a single operation during the follow-up period without any additional procedure. The bubble volume was estimated by measuring the semi-circular vertical height of the bubble in the eye under a slit-lamp microscope or a combined indirect ophthalmoscope. The PVR was graded using the 1983 criteria for PVRclassification^14^.

### Surgical procedures

Two experienced fundus surgeons (Jianhua Wu and Chao Feng) performed all the procedures under retrobulbar anaesthesia in this study. A standard three-port 23- or 25-gauge PPV (23G or 25G, depending on the surgeon's preference) was established 3.5–4.0 mm posterior to the limbus, and vitrectomy procedures were performed by using the Alcon Constellation surgical system (Alcon Laboratories, Fort Worth, TX) and a contact pan retinal wide-field visual system (Volk Miniquad XL Vit, Volk Optical, Inc.). After confirming that the perfusion head established posterior segment perfusion post vitreously, the core vitreous was first removed. Posterior vitreous detachment (PVD) was performed with triamcinolone acetonide staining if necessary. Intraoperative visual decisions regarding retinal mobility and fissure location were made, with or without the assistance of heavy water to flatten the posterior polar retina. In all cases, 360° episcleral depression was performed to thoroughly remove the vitreous at the edge of the retinal tear and the surrounding basal body to completely release the traction of all the vitreous around the retinal tear and reduce the potential traction of retinal thinning areas in other peripheral retinal areas unrelated to detachment. Next, balanced salt solution (BSS) perfusion fluid and air exchange, complete drainage of subretinal fluid at the edge of the hole, and attachment to the retina were performed, with retinal photocoagulation around the hole and degeneration area in 2–3 circles. Finally, sterile air was retained in the vitreous cavity in all cases, and the cannula was pulled out to observe the closure of the puncture incision. If the incision closure was inadequate, one needle was used for suture with an 8–0 absorbable suture. After the operation, the conjunctival sac was coated with tobramycin dexamethasone eye ointment and a sterile gauze cover. All patients underwent cataract extraction if the lens opacity was severe. Antibiotic eye drops were used for two weeks after surgery, and then the head-down or supine position was taken according to the location of the hole 24 h after surgery (when the hole occurred between 5 and 7 o'clock, the patient maintained the supine position to prevent residual liquid contact with the treated retinal hole).

### Statistical analysis

The data were statistically analysed by SPSS 25.0 (IBM, Armonk, New York, USA). Snellen visual acuity was converted into the logarithm of the minimum resolution angle (logMAR) score for statistical analysis. Visual acuity of hand movement was assigned 2.6 logMAR and counting finger acuity was assigned 2.3 logMAR^15^. The differences between preoperative and postoperative BCVA and IOP at different follow-up time points were compared by repetitive measurement deviation analysis. To identify the relevant preoperative factors that affect the initial anatomical success of RRD, we first conducted a univariate analysis to screen the variables with P < 0.1, in which the classified variables were tested by the chi-square test or Fisher’s exact probability method, and the independent-sample t tests or Mann–WhitneyU test were used for continuous variable data. Then, multivariate binary logistic regression (LR) (the forward LR method) was used to evaluate the effects of individual preoperative factors on the success rate of initial reduction. P < 0.05 was defined as a statistically significant difference.

## Results

A total of 92 cases (92 eyes) were included in this study, which comprised 42 males and 50 females, with an average age of 51.48 ± 10.95 (range: 22–75) years. As shown in Table 1, 86 eyes successfully reattachment after a single operation (93.5%), and 6 eyes failed in the first operation (6.5%). There were no significant differences between the successful and failure group in preoperatively baseline characteristics, including the gender, duration of symptoms, axial length, lens status, etc. (Table 1). However, the average age of the initial successful group was 52.31 ± 10.28 years, which was older than that of the failure group 39.50 ± 14.15 years, and this difference was statistically significant (P = 0.005, independent-sample t tests). Eleven eyes (12.8%) had retinal detachments referred to inferior quadrants in the initial reattachment success group. In contrast, in the failure group, there were three eyes (50%) with retinal detachment involving the inferior retina quadrants, and the difference between the two groups was statistically significant (P = 0.043, Fisher’s exact test). During the follow-up of our study, only 21 (27.6%) of 76 phakic eyes underwent phacoemulsification combined with intraocular lens implantation due to the progression of cataracts (Table 2). Univariate analysis selected four preoperative factors: age (P = 0.005, independent-sample t tests), disease symptom duration (P = 0.078, independent-sample t tests), whether inferior quadrants were involved in the retinal detachment (P = 0.043, Fisher’s exact test) and location of the retinal breaks (P = 0.065, Fisher’s exact test), which were related to the initial anatomic success of RRD. Multivariate logistic regression analysis showed that an older onset age of patients was a protective factor of the success of RD reattachment (odds ratio, 0.90; 95% confidence interval, 0.82 to 0.97; P = 0.010). However, retinal detachment of the inferior quadrants involved was a preoperative risk factor associated with the initial failure of retinal anatomical reattachment (odds ratio, 9.90; 95% confidence interval, 1.36 to 71.92; P = 0.023) (Table 3).

**Table 1:** Univariate Analysis Between Eyes with Success of Initial Reattachment and Eyes with Failure of Initial Reattachment After Primary Pars Plana Vitrectomy with Air Tamponade for Rhegmatogenous Retinal Detachment.

Clinical factors	Initial Reattachment		P value	Total n = 92
	Success(n = 86)	Failure(n = 6)		
Age (years, mean ± SD)	52.31 ± 10.28	39.50 ± 14.15	0.005	51.48 ± 10.98
**Sex, n (%)**				
Male	39 (45.3)	3 (50)	1.000†	42 (45.7)
Female	47 (54.7)	3 (50)		50 (54.3)
Symptom duration (days, mean ± SD)	10.17 ± 6.86	15.33 ± 6.74	0.078	10.51 ± 6.93
Axial length (mm, mean ± SD)	25.12 ± 2.20	26.02 ± 2.26	0.336	25.18 ± 2.20
High myopia, n (%)	30 (34.9)	3 (50)	0.663†	33 (35.9)
**Lens status, n (%)**				
Phakia	76 (88.4)	6 (100)	1.000†	82 (89.1)
Pseudophakia	10 (11.6)	0 (0)		10 (10.9)
Choroidal detachment, n (%)	13 (15.1)	1 (16.7)	1.000†	14 (15.2)
Preoperative IOP (mmHg, mean ± SD)	14.14 ± 3.38	13.17 ± 3.87	0.501	14.08 ± 3.40
Preoperative BCVA (logMAR units, mean ± SD)	1.43 ± 0.75	1.48 ± 0.48	0.865	1.43 ± 0.73
**Number of quadrants involved, n (%)**				
1 quadrant	29 (33.7)	3 (50)	0.463*	32 (34.8)
2 quadrants	48 (55.8)	3 (50)		51 (55.4)
3 quadrants	7 (8.1)	0 (0)		7 (7.6)
4 quadrants	2 (2.3)	0 (0)		2 (2.2)
Inferior quadrants involved, n (%)	11 (12.8)	3 (50)	0.043†	14 (15.2)
**Macula status, n (%)**				
On	31 (36.0)	2 (33.3)	1.000†	33 (35.9)
Off	55 (64.0)	4 (66.7)		59 (64.1)
PVR, n (%)				
None/A	34 (39.5)	2 (33.3)	0.885*	36 (39.1)
B	48 (55.8)	4 (66.7)		52 (56.5)
C1	4 (4.7)	0 (0)		4 (4.3)
Location of retinal breaks, n (%)				
Superior	81 (94.2)	4 (66.7)	0.065†	85 (92.4)
Inferior	5 (5.8)	2 (33.3)		7 (7.6)
**Number of retinal breaks, n (%)**				
Single	52 (60.5)	3 (50)	0.681†	55 (59.8)
Multiple	34 (39.5)	3 (50)		37 (40.2)
**Type of retinal breaks, n (%)**				
Horseshoe tears	48 (55.8)	2 (33.3)	0.458†	50 (54.3)
Atrophic holes	23 (26.7)	2 (33.3)		25 (27.2)
Horseshoe tears combined with atrophic holes	15 (17.4)	2 (33.3)		17 (18.5)
Lattice degeneration, n (%)	63 (68.5)	3 (50)	0.346†	66 (71.7)
Use of PFO, n (%)	33 (38.4)	3 (50)	0.675†	36 (39.1)
Number of photocoagulation spots, (spots, mean ± SD)	199.79 ± 72.72	183.17 ± 63.57	0.587	198.71 ± 71.96
Cryotherapy, n (%)	3 (3.5)	0 (0)	1.000†	3 (3.3)
**Gauge of vitrectomy instruments**				
23G	69 (80.2)	5 (83.3)	1.000†	74 (80.4)
25G	17 (19.8)	1 (16.7)		18 (19.6)

^†^Fisher’s exact test.

*Mann–Whitney U test.

IOP, intraocular pressure; BCVA, best correct visual acuity; logMAR, logarithm of the minimum angle of resolution; PVR, proliferative vitreoretinopathy; PFO, perfluoro-n-octane.

**Table 2:** Postoperative Clinical Characteristics of Patients.

Clinical factors	Initial Reattachment		P value	Total (n = 92)
	Success (n = 86)	Failure (n = 6)		
**Volume of air on first day postoperatively, n (%)**				
> 80%	40 (46.5)	0 (0)	0.004*	40 (43.5)
80%-50%	38 (44.2)	3 (50)		41 (44.6)
≤ 50%	8 (9.3)	3 (50)		11 (12.0)
Time of complete absorption of air (days, mean ± SD)	11.27 ± 1.97	8.83 ± 1.72	0.005*	11.11 ± 2.04
**Postoperative posture, n (%)**				
Prone	71 (82.6)	5 (83.3)	1.000†	76 (82.6)
Supine	15 (17.4)	1 (16.7)		16 (17.4)
**Lens status at the last follow-up, n (%)**				
Phakia	55 (64)	6 (100)	0.093†	61 (66.3)
Pseudophakia	31 (36)	0 (0)		31 (33.7)
Follow-up duration (months, mean ± SD)	8.15 ± 1.71	9.50 ± 1.38	0.061	8.23 ± 1.71
Final reattachment, n (%)	86 (100)	6 (100)	1.000†	92 (100)

^†^Fisher’s exact test.

*Mann–Whitney U test.

**Table 3:** Multivariate Logistic Regression of Risk Factors for Surgery Success.

Independent Variables	Odds Ratio	95% Confidence Interval	P value
Age (years)	0.90	0.82–0.97	0.010
Inferior quadrants involved (no vs. yes)	9.90	1.36–71.92	0.023

There was a statistically significant difference in the gas volume on the first day after the operation between the initial anatomic success group and the initial anatomic failure group (P = 0.004, Mann–Whitney U test), and the gas filling volume on the first day after surgery in the failure group was smaller than that in the success group. The average time of intraocular gas maintenance in the success group (11.27 ± 1.97 days) was longer than that in the surgical failure group (8.83 ± 1.72 days), and the difference between the two groups was statistically significant (P = 0.005, Mann–Whitney U test) (Table 2). Among the six eyes with initial reduction failure, 3 cases exhibited retinal re-detachment within one week after the operation: one case was due to the opening of the hole in the laser spot at the edge of the inferior original break, and the other 2 cases were due to the lack of a peripheral laser hole, which may in turn have been due to missed detection of the tear during vitrectomy, resulting in the recurrence of retinal detachment. In addition, another 3 of these six patients with failure had retinal detachment recurrence within three months after their initial surgery: 2 cases were due to PVR formation, and the other case was caused by a new tear adjacent to the primary breaks. Among all six patients with recurrence, four eyes underwent intraocular silicone oil filling during reoperation, and for two eyes, air was still chosen for intraocular tamponade (Table 4). Eventually, all the eyes were successfully reattached (100%) at the last follow-up.

**Table 4:** Summary of recurrent cases.

Cases	Sex (male/female)	Age (years)	Eye (right/left)	High myopia (yes/no)	Location of breaks	Number of breaks	Macular Status (on/off)	Inferior quadrants involved (yes/no)	Incision suture (yes/no)	Intraocular pressure on first day postoperatively (mmHg)	Time of recurrence	Causes of recurrence	Reoperation and intraocular tamponade agent	Tamponade time of silicone oil (months)	Final reattachment (yes/no)
1	Female	25	Left	No	Superior;2- o’clock meridian; anterior	Single	On	No	No	13.0	Within 3 months after PPV	PVR, preretinal membrane, subretinal membrane	Preretinal and subretinal membrane removing + photocoagulation + Silicone oil filling	3	Yes
2	Male	35	Right	Yes	Inferior; 8- o’clock meridian; equatorial	Single	Off	Yes	Yes	15.0	Within 1 week after PPV	The inferior peripheral retinal break is missed	Photocoagulation + Sterilized air filling	-	Yes
3	Female	48	Right	Yes	Inferior; 4–6 o’clock meridian; anterior	Multiple	Off	Yes	Yes	9.0	Within 1 week after PPV	The laser spots on the edge of the original break are opened again	Photocoagulation + Silicone oil filling	3	Yes
4	Male	54	Left	No	Superior; 10–12 o’clock meridian; anterior	Multiple	Off	No	No	8.0	Within 1 week after PPV	Small holes in the upper retinal degeneration area are missing	Photocoagulation + Silicone oil filling	3	Yes
5	Female	22	Left	No	Superior ;1–3 o’clock meridian; anterior	Multiple	On	No	No	15.0	Within 3 months after PPV	PVR, preretinal membrane	Preretinal membrane removing + Silicone oil filling	4	Yes
6	Male	53	Right	Yes	Superior; 11:30 o’clock meridian; equatorial	Single	Off	Yes	No	12.0	Within 3 months after PPV	A new break appears in adjacent position near to the primary hole of the temporal	Photocoagulation + Sterilized air filling	-	Yes

**Figure 1:**
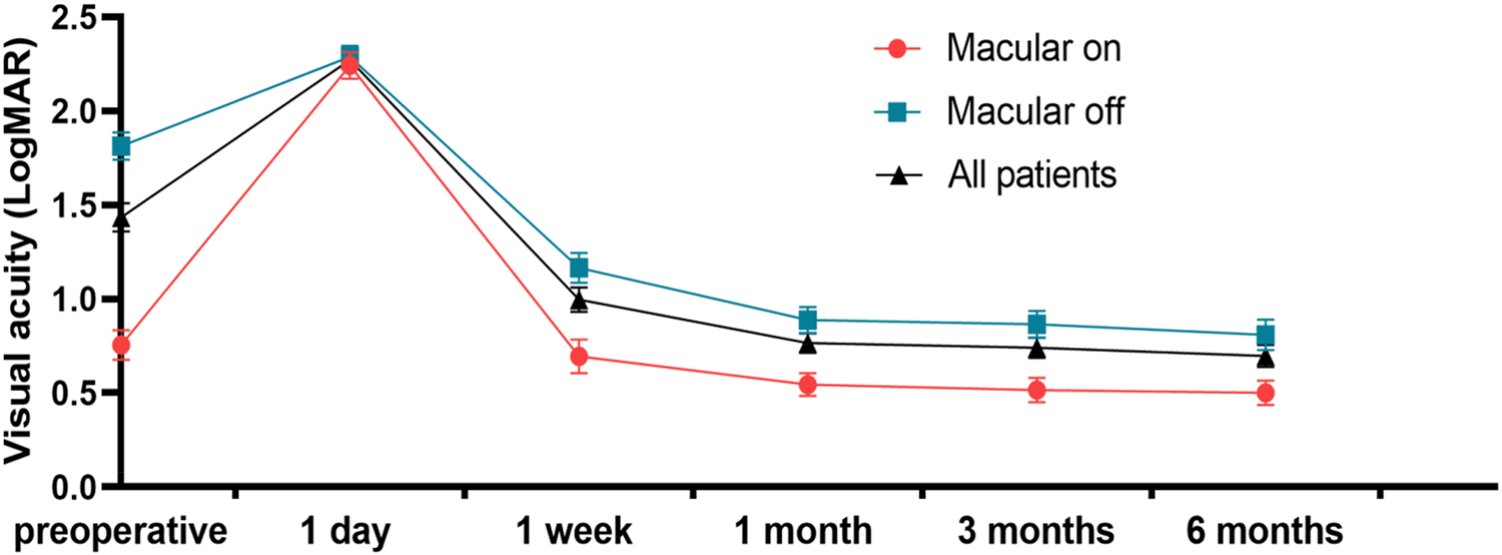
Visual outcome of patients undergone pars plana vitrectomy with air tamponade for rhegmatogenous retinal detachment.

The BCVA of all patients improved significantly from 1 week after surgery (preoperative BCVA 1.43 ± 0.73 logMAR, postoperative BCVA 1 day 2.27 ± 0.43 logMAR, 1 week 1.00 ± 0.63 logMAR, 1 month 0.77 ± 0.50 logMAR, 3 months 0.74 ± 0.52 logMAR, 6 months 0.70 ± 0.57 logMAR; P < 0.001, repetitive measurement deviation analysis). Except for the first day after surgery, the BCVA visual acuity of patients without macular involvement was better than that of patients with macular involvement. However, the best visual acuity improvement was similar between the two groups from 1 week after surgery (Fig. 1).

**Figure 2:**
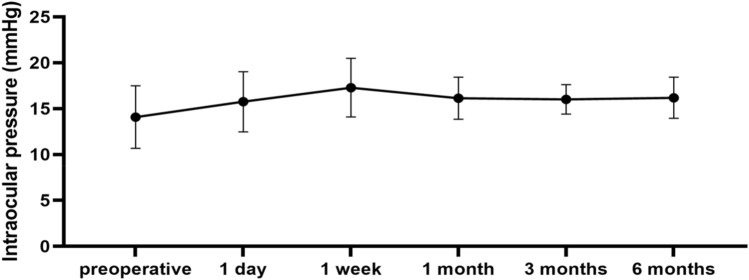
Intraocular pressure outcome of patients undergone pars plana vitrectomy with air tamponade for rhegmatogenous retinal detachment.

All patients' preoperative average IOP was 14.08 ± 3.40 mmHg, of which 10 eyes were below 10 mmHg. The postoperative IOP gradually recovered (1-day: 15.75 ± 3.28 mmHg, 1-week: 17.29 ± 3.20 mmHg, 1-month: 16.14 ± 2.29 mmHg, 3-month: 16.01 ± 1.60 mmHg, 6-month: 16.18 ± 2.24 mmHg; P < 0.001, repetitive measurement deviation analysis) (Fig. 2).

## Discussion

The selection of intraocular fillers after vitreoretinal surgery has always been a concern. The disadvantage of silicone oil filling is that it is necessary to perform secondary surgery and is accompanied by multiple complications^16^. Gas filling applies pressure to the retina mainly through the buoyancy and surface tension of the gas and prevents the liquid from entering the retina again through the hole before the retinal choroid forms a strong adhesion. Long-acting gases are commonly used clinically. However the absorption of long-acting gas is slow, making the surgical eye unable to restore vision in a short period, especially for patients with poor vision in nonsurgical eyes, which enormously affects their postoperative daily life. Meanwhile, the probability of postoperative cataracts is also significantly high in eye tamponades with expansion gas^17^. Several studies have also revealed that fluoride gases used in PPV, including sulfur hexafluoride (SF6), hexafluoroethane (C2F6), and octafluoropropane (C3F8), are some of the most potent greenhouse gases^18,19^. Moreover, the use of these long-acting gases in China is limitedfor legal reasons, and air has become one of the more straightforward options for treating RRD.

Unlike long-acting gases, air tamponade has many advantages, requiring no additional purchase, preservation, and dilution, and does not cause problems such as increased intraocular pressure, slow absorption, and ambient toxicity of gases. In addition, the use of air over expansile gases has the advantage of being much less damaging to the environment since expansile gases are potent greenhouse gases. More importantly, the faster absorption of air, the quicker recovery of visual function, and the early detection of failed retinal reattachment can reduce the incidence of cataracts^9,13^.

Previous studies have shown that retinal-choroid adhesions form within 24 h after retinal laser photocoagulation. Since then, the liquid in the vitreous cavity no longer enters the retina through the pores, and the pressure of the gas no longer seems necessary^8,20^. This provides a theoretical basis for the application of air as a filler in simple RRD. In the superior retinal detachment cases, there is no noticeable residue of subretinal fluid around the hole due to gravity, which affects the formation of retinal-choroidal adhesions. Therefore, early retinal-choroidal adhesion around the retinal hole can be achieved without long-term gas filling. Many studies have confirmed the effectiveness of air in such patients^9,11,21^. A similar adhesive effect can be obtained if the subretinal fluid at the edge of the hole is entirely drained and the hole edge is kept dry during the operation for inferiorly involved RRD. Martínez-Castillo et al. conducted three important vitrectomy studies on RRD with inferior fissures^10,20,22^. Their results showed that vitrectomy can treat RRD with inferior detachment even with air filling, and adequate drainage of subretinal fluid is an essential factor for the success of such cases. Zhou et al., a prospective study, reported that PPV combined with air filling could also achieve the same effect as C3F8 tamponade in treating inferior RRD^12^.

In our study, 92 cases (92 eyes) of RRD were treated with air filling, including 86 eyes (93.5%) with a single surgical reattachment. The reasons for the high rate of single surgical success in this study may be as follows. First, the included cases had a relatively short course of disease (the mean duration of symptoms was 10.51 ± 6.93 days), except for 4 cases whose PVR grade was C1, most of the PVR grades were B or A, and the retinal detachment in most cases only involved the superior quadrants (85 eyes, 92.4%). Second, we used a contact pan retinal wide-field visual system, which expanded the operating field and reduced the probability of iatrogenic breaks. Furthermore, in all cases, the peripheral vitreous was removed under episcleral depression, reducing the residual vitreous cortex. Third, at the end of the operation, we routinely sutured the puncture hole with 8–0 absorbable thread, avoiding air leakage, which ensured sufficient time for air filling in the eye. Therefore, it was observed that the average time for complete air absorption in the vitreous cavity in the case of successful reduction in a single operation in this study was 11.27 ± 1.97 days, which was longer than the 5–7 days reported in the literature^6,7^.

For noncomplicated RRD treated with vitrectomy combined with gas tamponade, there have been many previous studies on the prognostic factors of successful retinal reattachment after a single operation^11,12^. In our regression model, eyes with retinal detachment that involved the inferior quadrants (OR = 9.90; 95% Cl, 1.36 to 71.92; P = 0.023) appeared to be a preoperative risk factor affecting the success rate of retinal anatomical success after PPV and air filling. Compared with that in patients whose retinal detachment is in the upper quadrant, the air pressure may not be sufficient in eyes with involvement of the inferior quadrants, especially when this is combined with a lower tear in the retina. It is worth noting that volume of air in the eye after surgery is also very important, which may also affect the reduction of retinal detachment. In our study, patients with more than 80% intraocular air volume on the first postoperative day appeared to have a reduced rate of retinal detachment after primary surgery. Moreover, the exact onset time in patients with retinal detachment involving the lower quadrants may be longer, and the probability of PVR may also be higher than that of patients with retinal detachment involving the upper quadrants^23^.

Whether the presentation of age is related to the retinal reattachment success rate after vitrectomy for RRD is still controversial. Recently, Rao et al. found that patients who underwent a primary noncomplex RD repair, at an age of < 50 years with PPV ± SB, exhibited a higher odd of reoperation (OR: 1.46, 95% CI: 1.14–1.88) compared to SB only^24^. Patients > 50 years of age with PPV ± SB had a lower odd of reoperation (OR: 0.73, 95% CI: 0.63–0.84). Grosinger et al. also found that the age at presentation was a statistically significant protective factor in complicated RRD patients for reoperation^25^. In our multivariate model, age was also found to be a protective factor for initial successful retinal reattachment after PPV + air tamponade (OR: 0.90; 95% CI, 0.82–0.97; P = 0.010). PVR may be one of the main aspects explaining this result. Compared with elderly patients, younger patients have a more formed vitreous and stronger vitreous retinal adhesion, which makes it difficult to induce real posterior vitreous detachment during PPV^24,26^. Additionally, younger patients have a strong ability of tissue regeneration, which results in a high incidence of postoperative PVR^27^. Another reason may be related to the poor postural maintenance of younger patients. Because of work reasons or life activities, young patients tend to have poorer compliance than elderly patients, especially when their cases involve an inferior hole or lower quadrant retinal detachment. In contrast, elderly individuals may choose to avoid additional surgery, or age-related damage may prevent them from receiving further intervention and repeated surgery^25^.

In this study, 6 eyes with failed primary reattachment are shown in Table 4. All patients received retinal reattachment at the last follow-up. This detailed information prompts us to consider the following: (1) The opening of new or missed retinal tears is one of the leading causes of retinal re-detachment, and it is crucial to carefully look for retinal tears and use an appropriate laser to seal the retinal tears during the primary PPV. In a retrospective study by Li et al., a total of 59 eyes were treated with PPV + air, and in all cases, three to four rows of 360° prophylactic laser photocoagulation under scleral depression were applied. A primary anatomical success rate of 94.9% was achieved^9^. However, in our study, only 2–3 rows of laser photocoagulation were performed at the edges of the holes and degenerative areas, which may be one of the reasons for the recurrence of retinal detachment within one week in 3 of the cases. (2) Patients with proliferative membranes under the retina and traction of the retina should have these membranes removed as much as possible. (3) In patients with high myopia, atrophy of the fundus is obvious, especially when there is peripheral retinal degeneration or pigmentation, which is prone to the omission of some small pores, resulting in the recurrence of retinal detachment.

A secondary cataract is a common complication of RRD patients after PPV operation. The incidence of secondary cataracts in patients with PPV combined with long-acting gas filling within 3 months after the operation is approximately 18.8%, while 51.8% need cataract surgery within 6 months^17^, and 65% need surgery within 1 year^28^. During the follow-up of our study, only 21 (27.6%) of 76 phakic eyes underwent phacoemulsification combined with intraocular lens implantation due to the progression of cataracts, which was similar to the results reported by Li et al.^9^. The results suggested that PPV combined with air tamponade may reduce the risk of development or progression to cataracts.

Our study has several limitations: (1) It was a retrospective study. Only new cases of RRD were included, but there were no cases of giant tears, macular hole retinal detachment or RRD with a PVR grade above C1. In addition, there may be statistical bias due to the small number of recurrent cases. (2) Our study did not use long-acting gas fillers as a control because these gases were not permitted in China during the study period. (3) Although this was a continuous case series study, the study sample size was small; the follow-up time was short when the preoperative factors affecting the reduction were analysed; and some patients with poor vision or recurrent RRD may have had more or less frequent follow-up than patients with better vision. Therefore, the follow-up variables may have skewed our results. Nevertheless, we still believe that our findings are meaningful because we further verified the effectiveness of air in the treatment of simple retinal detachment with PPV and provided some reference factors for the selection of fillers in PPV surgery in such patients.

In conclusion, our study further confirms that vitrectomy plus air filling can obtain clinically satisfactory results in treating new primary RRD with a short duration of symptoms, a superior fissure and a PVR grade of C1 or below. Air as an intraocular filler can provide faster visual recovery and may reduce the risk of gas-related complications, such as cataract progression and high intraocular pressure. Our study also suggests that we should be careful when choosing air for intraocular filling in RRD patients with retinal detachment involving the lower quadrant and a younger age at onset. Undoubtedly, more prospective, randomized controlled trials yielding additional evidence are needed to further verify the exact efficacy of air as an intraocular filler in the future.
